# Leptospirosis manifested with severe pulmonary hemorrhagic syndrome successfully treated with veno-venous extracorporeal membrane oxygenation: A case report and literature review

**DOI:** 10.1097/MD.0000000000040942

**Published:** 2024-12-20

**Authors:** Xing-Cheng Zhang, Xi-Qun Lei, Yun Sun, Nan-Bing Shan

**Affiliations:** a Department of Critical Care Medicine, Fuyang Infectious Disease Clinical College of Anhui Medical University, Fuyang, Anhui Province, China; b Department of Critical Care Medicine, the Second Affiliated Hospital, Anhui Medical University, Hefei, Anhui Province, China.

**Keywords:** acute respiratory distress syndrome (ARDS), ECMO, leptospirosis, multi-organ failure, pulmonary hemorrhage

## Abstract

**Rationale:**

The mortality rate associated with pulmonary hemorrhage induced by leptospirosis is notably high. Available treatment modalities are limited, and their efficacy has not been fully demonstrated. Here, we present the case report of a patient with leptospirosis-induced pulmonary hemorrhagic syndrome. A 49-year-old male patient was admitted to the Surgical Ward of the Department of Intensive Care Medicine of Fuyang Infectious Disease Clinical College of Anhui Medical University. The patient had initially sought medical attention at a local hospital due to symptoms of fever persisting for 4 days and chest tightness accompanied by hemoptysis for 1 day.

**Patient concerns:**

We present the case report of a patient with leptospirosis-induced pulmonary hemorrhagic syndrome. Hemoptysis persisted in our patient during VV-ECMO, although we adjusted the heparin regimen to maintain an activated partial thromboplastin time target value of 50 to 55 seconds.

**Diagnoses:**

Leptospirosis-induced pulmonary hemorrhagic syndrome.

**Interventions:**

He was immediately intubated and mechanically ventilated and then transferred to our hospital for further medical intervention. Upon arrival at our hospital, he was treated with veno-venous extracorporeal membrane oxygenation (VV-ECMO). Consequently, he was administered penicillin and omacycline for anti-infective therapy. Anti-inflammatory agents, high-dose vasoactive drugs to enhance blood pressure, continuous renal replacement therapy, tracheal sputum aspiration, and ventilator-assisted ventilation were also administered as part of the treatment protocol.

**Outcomes:**

After treatment, his inflammation index was significantly decreased, the amount of pulmonary bleeding was reduced, his oxygenation ratio was improved, and the pulmonary lesions were absorbed. Consequently, he was discharged on the 34th day of hospitalization.

**Lessons:**

We successfully treated a case of leptospirosis pulmonary hemorrhagic syndrome using VV-ECMO combined with prudent anticoagulant therapy.

## 1. Introduction

Leptospirosis is an acute zoonotic infectious disease caused by pathogenic *Leptospira* species.^[1]^ Its main manifestations are leptospiremia in the early stage, organ damage and dysfunction in the middle stage, and allergic reactions in the late stage.^[2]^ Clinically, leptospirosis can be classified into several forms, including influenza-like, typhoidal, pulmonary hemorrhagic, jaundice hemorrhagic, renal-failure-associated, and meningitic, based on the specific organ systems affected.^[3]^ Leptospirosis is relatively rare in Fuyang City, Anhui Province. Its early symptoms lack distinct characteristics, and it frequently occurs as a co-infection with other diseases, making it challenging to differentiate from various infectious conditions. Consequently, misdiagnosis or missed diagnoses are common. Leptospirosis has a mean case fatality rate of 6.85%, with the highest risk for death among males 50 to 59 years of age.^[4]^ Of the disease-related complications, pulmonary hemorrhages are the major cause of mortality, accounting for 30% to 60% of the deaths.^[5–7]^ Despite the notably high mortality rate associated with pulmonary hemorrhages resulting from leptospirosis, the therapeutic landscape remains constrained by a lack of targeted treatment modalities, with the efficacy of current interventions yet to be comprehensively substantiated. Only a few cases successfully treated with extracorporeal membrane oxygenation (ECMO) have been reported.^[8]^ This article describes a case of severe pulmonary leptospirosis caused by *Leptospira interrogans*. The clinical data, imaging results, and disease outcomes are presented. This case of severe pulmonary leptospirosis was successfully treated by veno-venous ECMO (VV-ECMO). Accordingly, this article aims to highlight the effectiveness of ECMO in providing early support for patients with acute respiratory distress syndrome (ARDS) caused by diffuse alveolar hemorrhage (DAH), with the ultimate goal of improving the success rate of clinical treatments in similar cases.

## 2. Case presentation

### 2.1. Clinical presentation and patient diagnosis

**Figure 1.**
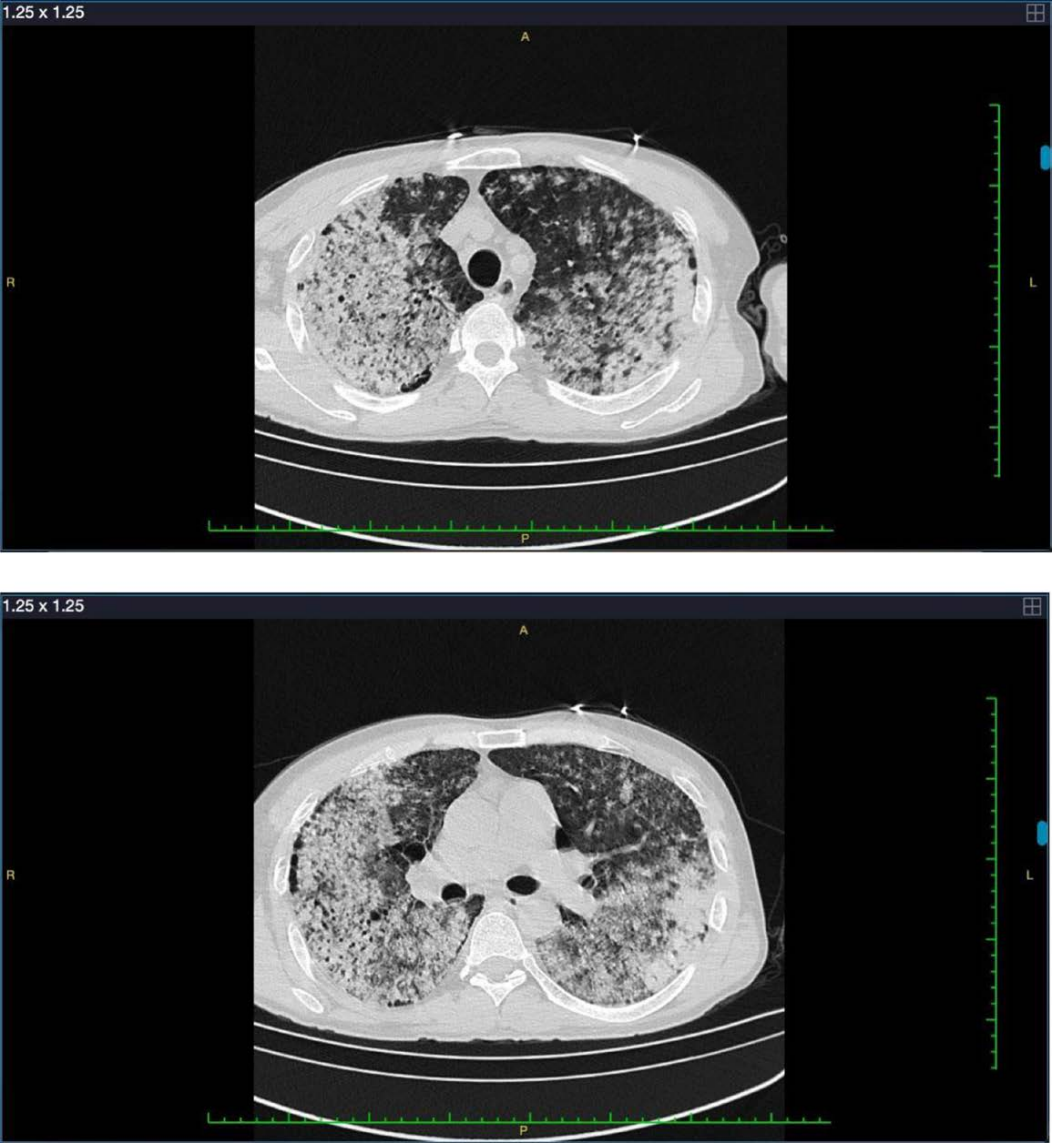
The chest computed tomography upon hospital admission on the first day of hospitalization showed diffusely increased-density.

A 49-year-old male patient with no prior medical history was admitted to a local hospital due to fever persisting for 4 days and chest tightness accompanied by hemoptysis for 1 day. According to the family account, the patient initially experienced fever, reaching a peak temperature of 39.4°C, along with chest discomfort and general fatigue, while working at a construction site near Qiandao Lake in Hangzhou City, Zhejiang Province, China, without an apparent cause. Subsequently, he self-administered antipyretic drugs (one 0.5 g acetaminophen tablet), which temporarily alleviated his symptoms, although occasional fever persisted. The patient urgently returned from Hangzhou to his hometown, Fuyang City, Anhui Province. Upon his return, he again experienced fever accompanied by chills, prompting his immediate presentation to the hospital for medical evaluation and treatment. Laboratory tests yielded the following results: white blood cell count of 11.53 × 10^9^/L, hemoglobin concentration of 100 g/L, platelet count of 53 × 10^9^/L, creatinine level of 111.1 µmol/L, procalcitonin level of 3.95 ng/mL, D-dimer level of 0.85 mg/L, K^+1^ concentration of 3.31 mmol/L, and Na^+1^ concentration of 133.1 mmol/L. Blood gas analysis revealed an oxygenation index (PaO_2_/FiO_2_ ratio, P/F) of 40 to 67 mm Hg, and chest computed tomography (CT) showed bilateral pneumonia (Fig. 1). Shortly after arriving at the hospital, the patient exhibited symptoms that included low blood pressure, dyspnea, cough, and significant hemoptysis (large quantity of bright red blood). Subsequently, the patient was intubated and received mechanical ventilation, hormone therapy, high doses of vasoactive drugs, organ support, and symptomatic treatment. Despite these interventions, his oxygenation index continued to struggle to maintain normal levels, even with pure oxygen support via mechanical ventilation. Consequently, he was transferred to the intensive care unit (ICU) surgical ward of Fuyang Infectious Disease Clinical College of Anhui Medical University for further treatment. Physical examination revealed a body temperature of 36.0°C, a respiratory rate of 19 breaths per minute, a heart rate of 135 beats per minute, and a blood pressure of 106/61 mm Hg (1 mm Hg = 0.133 kPa) with continuous infusion of norepinephrine at 0.80 μg/kg.min. The blood oxygen saturation of the patient was 84% with 100% oxygen supply. He was in a sedative and analgesic state, exhibiting slightly congested bilateral conjunctivae and normal systemic skin mucosa without apparent congestion. No lymph node enlargement was observed, and lip cyanosis was present. The patient had tracheal intubation in the mechanical ventilation mode with FIO_2_ at 100% and positive end-expiratory pressure (PEEP) at 18 cm H_2_O. Coarse breathing sounds were detected in both lungs, accompanied by prominent moist rales. The heart rate remained steady without any discernible pathological murmurs. The abdomen appeared flat and soft, with no palpable signs of pathology on either side. Therefore, the patient was presumptively diagnosed with DAH with severe ARDS.

### 2.2. Treatment

**Figure 2.**
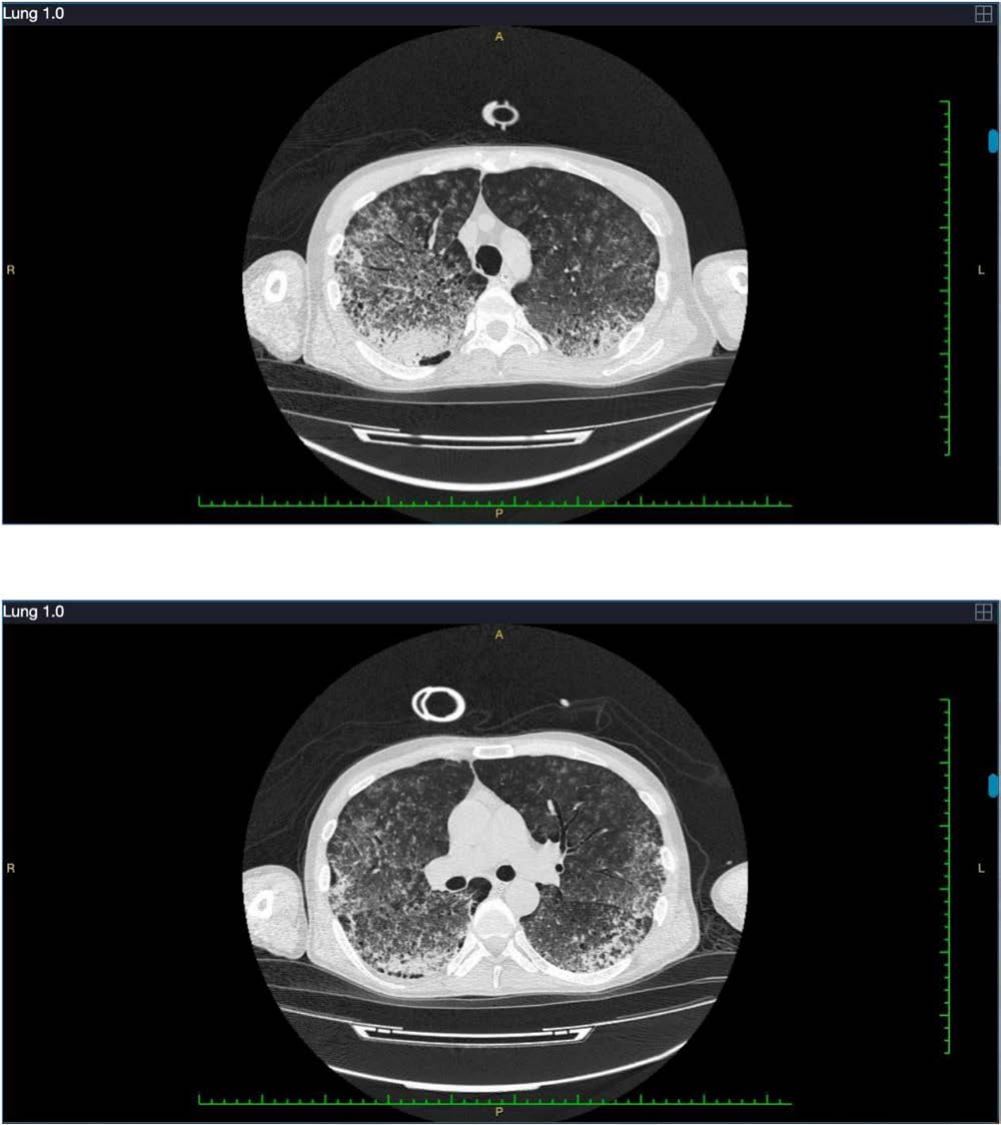
The chest computed tomography on the 9th day of hospitalization revealed patchy areas in both lungs.

The laboratory test results of the patient on the admission day were as follows: white blood cell count of 13.63 × 10^9^/L, hemoglobin concentration of 58.0 g/L, platelet count of 52 × 10^9^/L, neutrophil percentage of 92.9%, C-reactive protein level of 104.0 mg/L, lactate dehydrogenase activity of 265 U/L, α-hydroxybutyrate dehydrogenase activity of 181 U/L, creatine kinase activity of 97 U/L, creatinase isoenzyme activity of 14 U/L, creatinine level of 115 µmol/L, urea level of 9.9 mmol/L, uric-acid level of 392 µmol/L, interleukin-6 level of 243.70 pg/mL, B-type natriuretic peptide precursor (pro-BNP) level of 1769.00 pg/mL, procalcitonin level of 4.89 ng/mL, partial thromboplastin time of 46.7 seconds, fibrinogen level of 5.58 g/L, D-dimer level of 1.23 µg/mL, and antithrombin III level of 67.90%. The patient was administered piperacillin-tazobactam (4.5 g q8h) and moxifloxacin (400 mg qd) for anti-infection therapy, along with oseltamivir (75 mg q12h). He also received anti-inflammatory agents, hemostatic medications, albumin supplementation, plasma and red blood cell transfusions, stomach-protective measures, and therapies aimed at reducing phlegm. On the first day of hospitalization, the ventilator parameters were set to 18 cmH_2_O PEEP and 100% FiO_2_. Upon blood gas analysis, the P/F ratio was found to be 75 mm Hg. VV-ECMO was initiated to ensure adequate tissue oxygen supply and mitigate the risk of pulmonary barotrauma. The initial blood flow was set at 4.0 L/min, with a rotational speed of approximately 3000 r/min, 100% ECMO oxygen concentration, and an airflow rate of 4 L/min. The blood temperature was maintained at 36°C (Table 1). Bedside hemodialysis, tracheal sputum aspiration, hemostasis, mechanical ventilation, and other necessary treatments were administered concurrently. Metagenomic next-generation sequencing (mNGS) and relevant microbial cultures were conducted on the alveolar lavage fluid. On the third day of hospitalization, the mNGS analysis results revealed the presence of *Leptospira* (141 sequences with a relative abundance of 17.76% were detected) and *Streptococcus pneumoniae* (49 sequences with a relative abundance of 6.17%). On the fourth day of hospitalization, the patient’s serological test for leptospirosis indicated *serovar icterohaemorrhagiae.* Transthoracic echocardiography revealed normal left ventricular size and systolic function. The hemoculture and sputum culture were negative. Tests for sputum acid-fast bacilli and modified acid-fast bacilli were negative. Anti-HIV was negative. Anti-hepatitis C virus was negative. HBsAg, anti-HBs, anti-HBc were negative. The rapid influenza A/B/respiratory syncytial virus test was negative. Respiratory virus 6 subtypes were negative. In response to these findings, the antibiotic regimen was adjusted to penicillin (400,000 U q6h) and omacycline (0.1 g qd). Concurrent, continuous VV-ECMO therapy was maintained. On the seventh day of hospitalization, the patient exhibited elevated levels of inflammatory markers and fever (the maximum was 40.5°C). His white blood cell count was 47.43 × 10^9^/L, and the total bilirubin level was 42.1 µmol/L. Empirically, vancomycin (1 g Q12h) was administered, accompanied by liver-protective therapy. On the ninth day of hospitalization, the patient exhibited a decreased inflammation index and increased oxygenation compared with the previous levels. Subsequent chest CT reexamination revealed that the pulmonary hemorrhage and infection were reduced in both lungs, with significantly reduced lesion size and numbers compared with those in the earlier images (Fig. 2). Having met the criteria for withdrawal, ECMO was discontinued. However, continuous renal replacement therapy (CRRT) and mechanical ventilation were continued as part of the ongoing treatment protocol. On the 10th day of hospitalization, the results of the alveolar lavage fluid examination indicated a galactomannan (GM) level of 2.019 µg/L. In response, esaconazole (200 mg q8h) was introduced for antifungal treatment. The patient underwent fiberoptic bronchoscopy once more, revealing diffuse endobronchial hemorrhage. Sputum samples were collected for culture and fungal GM testing. On the 11th day of hospitalization, the laboratory reported the sputum culture results, which indicated the presence of *Klebsiella pneumoniae* with a negative extended-spectrum beta-lactamase status. Consequently, imipenem cestatin (1 g q8h) was included in the treatment regimen. On the 12th day of hospitalization, the patient experienced recurring hemoptysis. Thus, the dose of the penicillin G was adjusted to 3.2 million units every 6 hours (q6h). On the 18th day of hospitalization, treatment with imipenem cilastatin at 1 g q8h was replaced with cefoperazone sodium and sulbactam 3 g q8h.

**Table 1:** Extracorporeal membrane oxygenation (ECMO) settings.

ECMO day	1	2	3	4	5	6	7	8	9
Blood flow (L/min)	3.98	3.99	3.74	3.61	3.74	3.62	3.44	3.32	3.35
Gas flow (L/min)	4	4	3.5	4.0	3.5	4.5	3	3	1.5
F_D_O_2_	1	1	1	1	1	1	1	1	1
Motor RPM	3030	3030	2970	3000	3000	3000	2845	2845	2845

Note: The ECMO equipment was from MAQUET.

Abbreviation: RPM = revolution per minute.

## 3. Results

**Figure 3.**
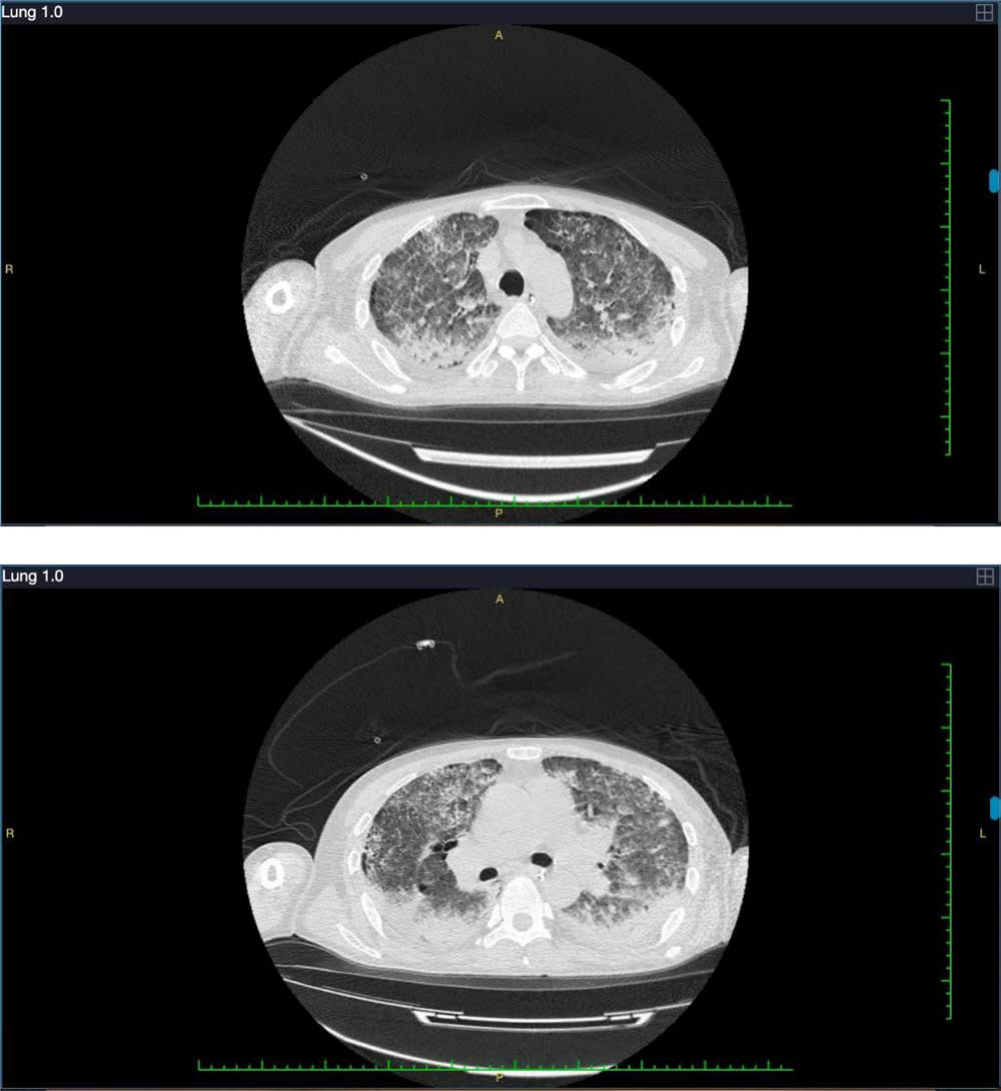
The chest computed tomography on the 22nd day of hospitalization showed patchy, flaky, strip-like increased-density shadows in both lungs, with obvious absorption.

**Figure 4.**
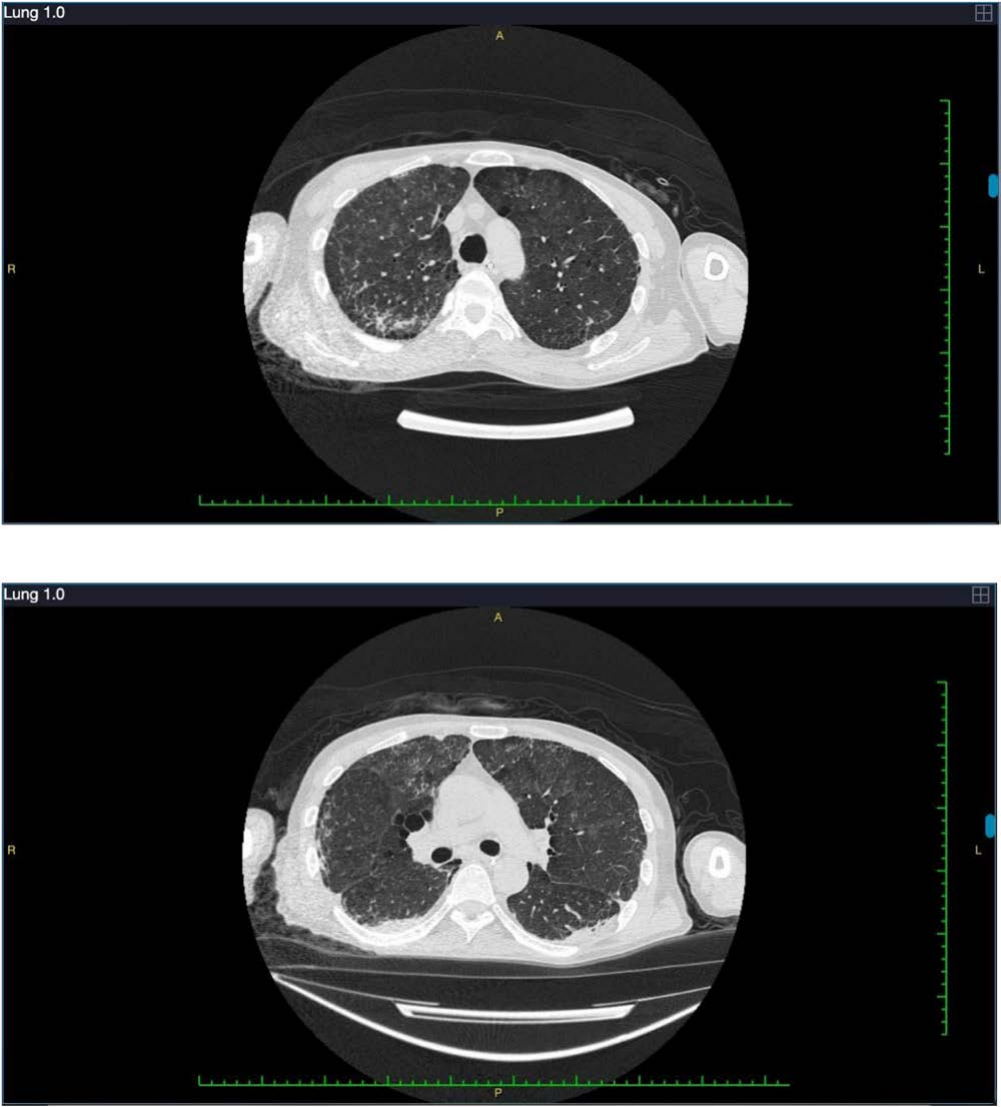
The chest computed tomography on the 29th day of hospitalization showed increased lung density, indicating a smaller lesion area than the 1 observed on the 22nd day of hospitalization.

On the 21st day of hospitalization, the patient had no fever and a stable oxygenation index, as well as reduced internal airway bleeding and a decreased infection index compared to the previous days. After removing the ventilator, the oxygenation index of the patient remained satisfactory, and the tracheal intubation was removed. On the 22nd day of hospitalization, the patient used a high-flow humidification device for oxygen therapy at 50% FIO_2_. Blood gas analysis revealed an oxygen partial pressure of 131 mm Hg and an oxygenation index of 262 mm Hg. Chest CT images displayed significant absorption of the lesions (Fig. 3). The levels of inflammatory markers were near normal, and liver and kidney function was improved (Table 2). The condition of the patient remained stable, prompting the initiation of rehabilitation activities, including bedside sitting and standing exercises. On the 29th day of hospitalization, the chest CT revealed that the lesions had essentially resolved and improved (Fig. 4). The patient reported no discomfort, such as hemoptysis or sputum. Consequently, he was discharged on the 34th day of hospitalization.

**Table 2:** Evolution of patient’s clinical parameters during the hospital stay.

Chronology	Day 1	Day 2	Day 3	Day 9	Day 11	Day 20	Day 34
Arterial blood gas							
pH	7.24	7.40	7.45	7.33	7.37	7.40	7.38
PaCO_2_ (mm Hg)	46.2	50.7	35.2	33.0	32.7	33.4	35.0
PaO_2_ (mm Hg)	75	85	137	203	31	140	130
PaO_2_/FiO_2_	75	189	137	405	431	350	321
SaO_2_ (%)	92.6	96.2	99.0	99.5	99.4	99.0	99.0
HCO_3_^-^ (mmol/L)	20.0	31.7	24.2	17.3	26.4	20.9	23.5
Lac (mmol/L)	7.8	2.9	1.6	0.7	1.0	0.3	1.0
Biochemistry							
AST (U/L)	21	2480	1297	10	25	9	33
ALT (U/L)	9	959	875	24	17	7	69
TBil (µmol/L)	20.6	28.9	42.4	14.8	25.9	16.9	5.3
Cr (µmol/L)	115	131	138	95	115	116	78
Urea (mmol/L)	9.9	8.6	9	9.4	9.6	24.3	8.4
Hb (g/L)	58	53	89	106	81	71	107
WBC (×10^9^/L)	13.63	17	17.71	20.62	11.15	4.23	7.61
PLT (×10^9^/L)	105	51	74	56	73	163	346
CRP (mg/L)	142.2	104	157.0	19	60.2	24.3	8.3

Abbreviations: ALT = alanine aminotransferase, AST = aspartate aminotransferase, Cr = creatinine, CRP = C-reactive protein, Hb = hemoglobin, Lac = lactate, PLT = platelet count, TBil = total bilirubin, WBC = white blood cell count.

### 3.1. Follow-up

**Figure 5.**
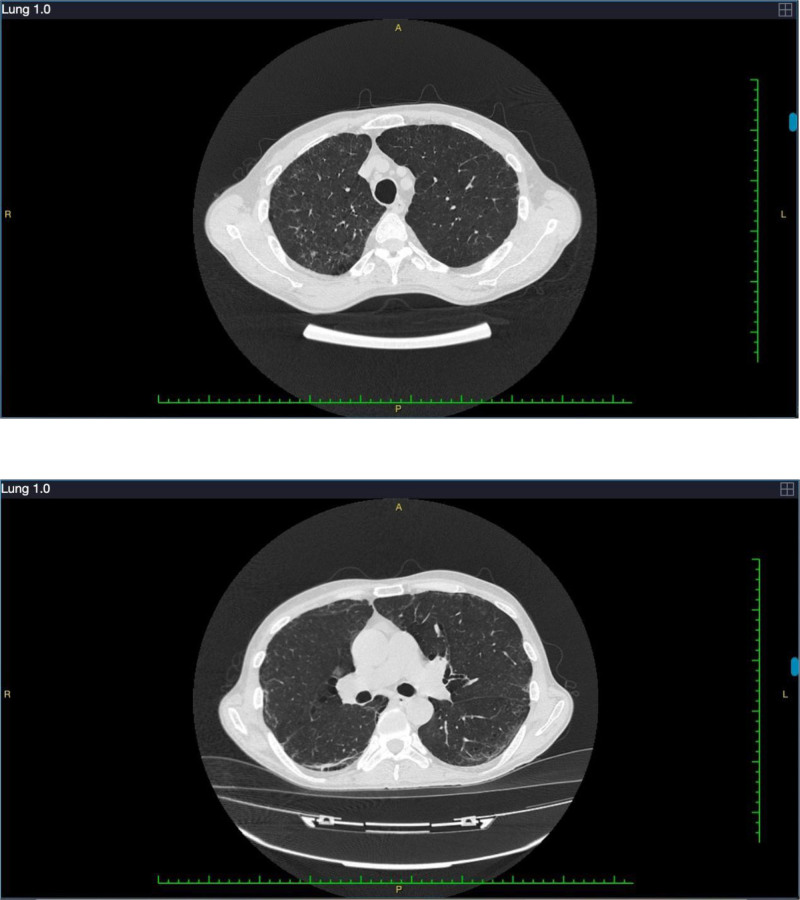
The chest computed tomography on the 17th day after discharge showed that lesion absorption had improved.

On the 17th day after discharge, the patient underwent a chest CT examination in the outpatient department of our hospital, which showed that the absorption of lung lesions had basically improved (Fig. 5).

## 4. Discussion

*Leptospira* can be divided into *Leptospira interrogans* and *Leptospira biflexa*. Of these 2 groups, *Leptospira interrogans* is pathogenic. Leptospirosis is prevalent in *Zhejiang* Province, China, and is particularly concentrated in the mountainous regions of central and southern *Zhejiang*. Peak leptospirosis epidemics occur during summer and autumn, and the incubation period typically ranges from 7 to 14 days.^[9]^ The disease often manifests in the lungs, affecting up to 70% of cases worldwide,^[10]^ and can lead to complications, such as leptospirosis pulmonary hemorrhagic syndrome (LPHS), and ARDS. LPHS is increasingly recognized as a severe manifestation of the disease, with a mortality rate exceeding 50% worldwide.^[11]^

The patient’s serological test for leptospirosis indicated serovar icterohaemorrhagiae. This serovar is highly virulent compared to other serovars of *Leptospira interrogans*. It is particularly associated with severe forms of leptospirosis, such as Weil disease, and is characterized by jaundice, renal failure, and hemorrhage. The pathogenesis involves the ability of the bacterium to invade and spread throughout the host’s tissues, leading to systemic inflammation and organ damage. The infection typically begins with flu-like symptoms, such as fever, headache, and muscle pain. In severe cases, it progresses to Weil disease, with symptoms including jaundice (yellowing of the skin and eyes), renal impairment, and bleeding disorders.^[12]^ Pulmonary hemorrhage may also occur, which is a severe complication and a significant cause of death in leptospirosis patients.^[13]^ *Leptospira interrogans* serovar Icterohaemorrhagiae is serologically distinct, which allows it to be differentiated from other serovars through serological testing. The bacterium’s outer membrane proteins and lipopolysaccharides play a role in its virulence and are targets for serological identification.^[14]^ This serovar is commonly found in urban settings and is often associated with infected rodents, particularly rats, which act as the primary reservoirs and shed the bacteria in their urine. Human infection typically occurs through direct or indirect contact with contaminated water sources.

The residence and disease-onset time of the patient were consistent with typical leptospirosis patterns. Additionally, the primary complaint and classification aligned with the characteristics of pulmonary hemorrhagic leptospirosis. The main manifestation of this disease is DAH, characterized by a rapid onset, swift progression, dangerous nature, and potentially fatal hemoptysis. The mechanism of hemorrhage caused by *Leptospira interrogans* remains incompletely clarified. Pulmonary injury might be induced by an unspecified leptospiral toxin, leading to endothelial damage in pulmonary capillaries, or by host immune responses.^[15–17]^ Recent studies have also suggested that it may be related to hemolysin,^[13]^ the platelet-activating factor acetylhydrolase,^[18]^ or tumor necrosis factor-α. The pathological basis of pulmonary hemorrhagic leptospirosis includes leptotoxin-induced microcirculation disturbances and the infiltration of numerous red blood cells into the alveoli. This process leads to capillary congestion, hemorrhage, pulmonary diffuse hemorrhage, the obstruction of alveolar oxygen diffusion, and subsequently, ARDS. This pathophysiological mechanism elucidates the development of ARDS in the presented case. Consequently, the patient required endotracheal intubation and mechanical ventilation to maintain vital signs upon admission to the local hospital.

China widely employs penicillin, glucocorticoids, and mechanical ventilation to treat pulmonary hemorrhagic leptospirosis. ECMO may be considered when oxygenation cannot be maintained via mechanical ventilation. In our case, the patient was treated with the broad-spectrum antibiotic piperacillin sodium tazobactam (4.5 g q8h) + moxifax (400 mg qd) + oseltamivir (75 mg q12h) for infection control immediately after hospital admission. Subsequently, his dyspnea symptoms worsened, and his P/F ratio progressively deteriorated under ventilator support. Emergency ECMO intervention was performed. The oxygen saturation of the patient was 74 to 80% before initiating VV-ECMO and improved to 98 to 100% within minutes after initiating the intervention. Sputum samples were subjected to mNGS instantly, and the genetic test report confirmed that the pathogen was *Leptospira interrogans*. The antibiotic regimen was promptly adjusted to penicillin (400,000 U q6h) + omacycline (0.1 g qd), supplemented with hormones against the Hershey reaction. CRRT was promptly applied when the patient showed anuria and increased creatinine levels. CRRT intervention was employed to eliminate endotoxins, inflammatory mediators, metabolites, and substantial amounts of myoglobin. Following the adjustment of the medication regimen, the laboratory test results and imaging results of the patient showed significant improvements. The levels of inflammatory markers decreased markedly, the pulmonary blood loss was reduced, the P/F ratio was improved, and the lung lesions were substantially absorbed. The treatment efficacy was satisfactory, and the patient’s prognosis was good.

The key to the successful treatment of this case lies in the following 2 aspects: clear early diagnosis and timely and effective ECMO intervention.

The traditional serological method is the gold standard for detecting pathogenic *Leptospira* strains. However, its operation is intricate, involving complex experimental technology, and its routine implementation is limited. Conversely, mNGS has the advantages of being high throughput and highly accurate and is widely used in personalized treatment, such as in cases of tumor diagnosis, the detection of pathogenic genes, the diagnoses of genetic diseases, noninvasive prenatal genetic testing, and pre-implantation genetic screening and diagnosis.^[19–21]^ Its role has become increasingly prominent in the detection of pathogenic microorganisms. In our case, leptospirosis was definitively diagnosed by applying this technique. Twelve recent case reports have described the use of VV-ECMO in patients with leptospirosis complicated by ARDS and hemorrhagic pneumonia.^[22–24]^ In these cases, only 1 patient succumbed to the condition,^[22–24]^ a considerably lower mortality rate than the reported figure of > 50% among patients without ECMO support in the ICU.^[25]^ This observation indicates that ECMO can significantly enhance prognosis and reduce mortality in patients experiencing severe respiratory failure due to pulmonary hemorrhage. In addition, the duration of ECMO in surviving patients is 2 to 18 days, usually 1 week.^[13,26]^ This duration aligns with the ECMO support period observed in our case. Plasmapheresis was not considered in our case due to the lack of proven efficacy. ECMO also serves as a symptomatic treatment for respiratory failure, addressing persistent hypoxemia that can potentially result in multi-organ dysfunction. Its adoption is a relatively recent development compared to plasma exchange and has garnered significant attention, particularly in cases of leptospirosis-associated pulmonary bleeding. Therefore, we prefer using ECMO in severe cases of pulmonary hemorrhage and apply it early.

Hemoptysis persisted in our patient during VV-ECMO, although we adjusted the heparin regimen to maintain an activated partial thromboplastin time target value of 50 to 55 seconds. Therefore, the patient received a substantial transfusion of blood products (16 units of red blood cells and 4200 mL of fresh-frozen plasma) with the continuous administration of pituitin. The patient in this case was difficult to wean from the ventilator because of persistent alveolar bleeding after ECMO withdrawal, which has been reported only infrequently in the literature. A local citric acid anticoagulation regimen was implemented during the CRRT period to mitigate further alveolar bleeding. Simultaneously, the penicillin dosage was adjusted to 3.2 million U q6h. Eventually, the patient was successfully extubated. Methylprednisolone has previously been used to treat pulmonary hemorrhage. However, no significant reduction in mortality has been observed in mechanically ventilated patients.^[27]^ Trivedi et al reported the efficacy of cyclophosphamide and plasma exchange in the treatment of leptospirosis pulmonary hemorrhage. However, severely ill patients may not tolerate transient hypoxemia associated with plasma exchange.^[28]^ High-frequency oscillatory ventilation can effectively treat pulmonary hemorrhage, albeit with the potential drawback of significant hemodynamic instability.^[29]^ Another symptomatic treatment involves the application of aminocaproic acid. In 2017, Pardinas et al^[30]^ first reported the continuous infusion of aminocaproic acid and adjustment of the heparin regimen to prevent alveolar bleeding in LPHS patients during continuous ECMO. Although this approach led to significant improvement in symptoms and a notable decrease in the need for blood products, the exact role of aminocaproic acid remained unclear. Strategies aimed at preventing further hemorrhage in patients with active bleeding while on ECMO include the use of fresh-frozen plasma, vitamin K, aprotinin, and recombinant activated factor VII. Additionally, it is recommended to avoid the concurrent use of heparin and instead utilize only nafamostat mesylate as a regional anticoagulant.^[31–33]^

## 5. Conclusion

Leptospirosis is a rare infectious disease in Fuyang City, Anhui Province, emphasizing the importance of conducting early etiological detection for suspected cases. In cases involving severe respiratory failure, immediate consideration of ECMO and other extracorporeal life-support methods is vital as an emergency treatment option. Such interventions can significantly reduce the mortality rate among patients severely affected by leptospirosis. Finally, we also summarized the current management strategies of persistent alveolar hemorrhage in patients with leptospirosis, hoping to provide a reference and help for clinicians in the treatment of leptospirosis.

## Author contributions

**Conceptualization:** Xing-Cheng Zhang.

**Data curation:** Xing-Cheng Zhang.

**Funding acquisition:** Yun Sun, Nan-Bing Shan.

**Methodology:** Xi-Qun Lei, Nan-Bing Shan.

**Project administration:** Yun Sun.

**Resources:** Xi-Qun Lei, Yun Sun.

**Supervision:** Xi-Qun Lei, Nan-Bing Shan.

**Writing – original draft:** Xing-Cheng Zhang.

**Writing – review & editing:** Yun Sun, Nan-Bing Shan.
